# Supplementary material for: Development of the Italian Clinical Practice Guidelines on Bariatric and Metabolic Surgery: Design and Methodological Aspects
Source: Nutrients. 2022 Dec 30;15(1):189. doi: 10.3390/nu15010189 (PMC9823862; doi:10.3390/nu15010189)
Supplement: Supplementary file 1 [file nutrients-15-00189-s001.zip › nutrients-2111633-supplementary.pdf]

Supplementary Material:

Table S1 – Characteristics and tasks of all panelists

N	Name	Profession	Role	Scientific society
1	*Maurizio De Luca*	*Surgeon*	*Coordinator*	*SICOB*
2	*Marco Antonio Zappa*	*Surgeon*	*Member*	*SICOB*
3	*Eugenia Romano*	*Psychologist*	*Member*	*EASO*
4	*Giuseppe Navarra*	*Surgeon*	*Member*	*SICOB*
5	*Francesco Maria Carrano*	*Surgeon*	*Member*	*SICOB*
6	*Monica Zese*	*Surgeon*	*Co-coordinator*	*SICOB*
7	*Antonio Vitiello*	*Surgeon*	*Member*	*SICOB*
8	*Ugo Bardi*	*Surgeon*	*Member*	*SICOB/ACOI*
9	*Giovanni Casella*	*Surgeon*	*Member*	*SICOB*
10	*Sonja Chiappetta*	*Surgeon*	*Member*	*SICOB*
11	*Angelo Iossa*	*Surgeon*	*Member*	*SICOB*
12	*Emanuele Soricelli*	*Surgeon*	*Member*	*SICOB*
13	*Alessandro Martinino*	*MD, Research Fellow*	*Member*	*SICOB*
14	*Giacomo Piatto*	*Surgeon*	*Co-coordinator*	*SICOB/IFSO*
15	*Marco Raffaelli*	*Surgeon*	*Member*	*SICOB*
16	*Matteo Monami*	*Diabetologist*	*Methodologist*	*SID*
17	*Marco Chianelli*	*Endocrinologist*	*Member*	*AME*

N	Name	Profession	Role	Scientific society
18	*Roberto Serra*	*Obesiologist*	*Member*	*SIO*
19	*Gerardo Medea*	*General practitioner*	*Member*	*SIMG*
20	*Simone Rugolotto*	*Pediatrician*	*Member*	*SIP*
21	*Fausta Micanti*	*Psychiatrist*	*Member*	*SICOB*
22	*Maria Grazia Carbonelli*	*Obesiologist*	*Member*	*ADI*
23	*Valentina Lorenzoni*	*Economist*	*Member*	*SIS*
24	*Iris Maria Caterina Zani*	*Patients' association*	*Member*	-

SICOB: Società Italiana di Chirurgia dell'Obesità (Italian Society of Obesity Surgery)**; ACOI:** Associazione Chirurghi Ospedalieri (Hospital Surgeons Association)**; IFSO:** International Federation for the Surgery of Obesity and Metabolic Disorders**; SID:** Società Italiana di Diabetologia (*Italian Society for the Study of Diabetes*)**; AME:** Associazione Medici Endocrinologi (Endocrinologist Association)**; SIO:** Società Italiana dell'Obesità (Italian Society of Obesity)**; SIMG:** Società Italiana di Medicina Generale e delle Cure Primarie (Italian Society of General Medicine and Primary Care); **SIP:** Società Italiana di Pediatria (Italian Society of Pediatrics)**; ADI**: Associazione Italiana di dietetica e Nutrizione Clinica (Italian Association of Dietetics and Clinical Nutrition)**; SIS:** Società Italiana di Statistica (Italian Society of Statistics)**.**

Table S2 – PICO not reaching consensus and requiring a second Delphi round; *second round.

N	PICO	Disagreement (score 1–2)	Agreement (score 3–5)	Approval
**1**	***In patients with BMI≥ 30 kg/m², is bariatric and metabolic surgery preferable to non-bariatric/metabolic surgical treatments, for the treatment of active malignancies?***	**83%**	**17%**	❌
**2**	***In patients with BMI≥ 30 kg/m² and metabolic associated fatty liver disease, is bariatric/metabolic surgery preferable to non-bariatric and metabolic surgical treatments, for the treatment of fatty liver disease?***	**75%**	**25%**	❌
	In patients with BMI≥ 30 kg/m² and dumping syndrome after bariatric/metabolic surgery, is **educational therapy** preferable to **generic advices**, for the treatment of dumping syndrome?	66%	34%	❗

3				
**3***	***In patients with BMI≥ 30 kg/m^2 and dumping syndrome after bariatric/metabolic surgery, is medical nutritional therapy preferable to non-medical nutritional therapy, for the treatment of dumping syndrome?***	**75%**	**25%**	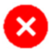
**4**	***In patients with BMI< 30 kg/m^2, is bariatric and metabolic surgery preferable to non-bariatric/metabolic surgical treatments, for the treatment of overweight?***	**87%**	**13%**	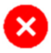
5	In patients with BMI≥ 30 kg/m2 with indication to bariatric/metabolic surgery, the pre-operative screening with specifically-designed questionnaire of obstructive sleep apnea is preferable to non-screening, for reducing peri-operative complications?	34%	66%	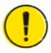
6	In patients with BMI< 30 kg/m2 and diabetes, is bariatric and metabolic surgery preferable to non-bariatric/metabolic surgical treatments, for the treatment of diabetes?	66%	34%	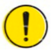
**6**	***In patients with BMI< 30 kg/m^2 and uncontrolled diabetes, is bariatric and metabolic surgery preferable to nonbariatric/metabolic surgical treatments, for the treatment of diabetes?***	**75%**	**25%**	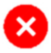

Table S3 – Main characteristics of studies for being included in metanalyses for each approved (≥ 7 points) outcome.

N*	Outcome	Type of study	Main inclusion criteria
1.1, 2.1, 3.1, 4.1, 5.1	Diabetes remission	RCT	Patients aged 18+ years, with obesity and diabetes (or subgroups with diabetes); studies performed on patients with BMI 3034.9, >35, and >40 Kg/m^2 and duration≥ 52 weeks[a,b,c]; diabetes remission[1] will be evaluated yearly whenever possible, and at endpoint.
1.2, 2.2, 3.2, 4.2, 5.2	Improvement of glycometabolic control	RCT	Patients aged 18+ years, with obesity +/- diabetes; studies performed on patients with BMI 30-34.9, >35, and >40 Kg/m^2 and duration≥ 52 weeks. HbA1c; FPG; lipid profile; blood pressure at endpoint[a,b,c].

1.3, 2.3, 3.3, 4.3, 5.3	Decrease of body weight	RCT	Patients aged 18+ years, with obesity +/- diabetes; studies performed on patients with BMI 30-34.9, >35, and >40 Kg/m^2 and duration≥ 52 weeks. BMI, percentage of weigh lost, and percentage of fat mass at endpoint[a,b,c].
1.4, 2.4, 3.4, 4.4, 5.4	Reduction of macrovascular complications	RCT	Patients aged 18+ years, with obesity +/- diabetes; studies performed on patients with BMI 30-34.9, >35, and >40 Kg/m^2 and duration≥ 52 weeks. Nonfatal myocardial infarction, nonfatal stroke e cardiovascular mortality (composite endpoint) at endpoint[a,b,c].
1.5, 2.5, 3.5, 4.5, 5.5	Reduction of all-cause mortality	RCT	Patients aged 18+ years, with obesity +/- diabetes; studies performed on patients with BMI 30-34.9, >35, and >40 Kg/m2 and duration≥ 52 weeks[a,b,c]. Mortality at endpoint.
1.6, 2.6, 3.6, 4.6, 5.6	Improvement of quality of life	RCT	Patients aged 18+ years, with obesity +/- diabetes; studies performed on patients with BMI 30-34.9, >35, and >40 Kg/m2 and duration≥ 52 weeks. Quality of life measured with any questionnaire at endpoint[a,c].
4.7, 5.7	Hypertension remission	RCT	Patients aged 18+ years, with obesity and at least one comorbid condition; studies performed on patients with BMI>35 or BMI>40 Kg/m^2, and duration≥ 52 weeks. Remission is defined as normal blood pressure values without any antihypertensive drugs[2].
4.8, 5.8	Obesity-related complications remission	RCT	Patients aged 18+ years, with obesity and at least one comorbid condition; studies, with duration≥ 52 weeks, performed on patients with BMI>35 or BMI>40 Kg/m^2. Remission for obstructive sleep apnea is defined as 1) C-PAP discontinuation[3], 2) symptoms remission (with specific questionnaires) or 3) Apnea-Hypopnea Index< 5 events/h at polysomnography[3] . Remission of dyslipidemia is defined as normal values of cholesterol without drugs[2]. Remission for hypertension[2] is defined for normal blood pressure values without any antihypertensive drugs[a,b,c].
1.7, 2.7, 3.7, 4.9, 5.9	Perioperative mortality	RCT	Patients aged 18+ years, with obesity +/- diabetes; studies, with duration≥ 52 weeks, performed on patients with BMI 30-34.9, >35, and >40 Kg/m^2. Mortality during surgical procedures or hospital stay.

N*	Outcome	Type of study	Main inclusion criteria
1.8, 2.8, 3.8, 4.10, 5.10	Perioperative surgical complications	RCT	Patients aged 18+ years, with obesity +/- diabetes; studies, with duration≥ 52 weeks, performed on patients with BMI 30-34.9, >35, and >40 Kg/m^2. Surgical complications during surgical procedures or hospital stay.

1.9, 2.9, 3.9, 4.11, 5.11	Serious adverse events (surgical and nonsurgical)	RCT	Patients aged 18+ years, with obesity +/- diabetes; studies, with duration≥ 52 weeks, performed on patients with BMI 30-34.9, >35, and >40 Kg/m^2. Any serious adverse events during surgical procedures or during follow-up at endpoint.
6.1	Obesity-related complications remission	RCT	Patients aged 13-17 years, with obesity and at least one comorbid condition; studies, with duration≥ 52 weeks, performed on patients with BMI>35 or BMI>40 Kg/m^2. Remission for obstructive sleep apnea is defined as 1) C-PAP discontinuation[3], 2) symptoms remission (with specific questionnaires) or 3) Apnea-Hypopnea Index< 5 events/h at polysomnography[3]. Remission of dyslipidemia is defined as normal values of cholesterol without drugs[2]. Remission for hypertension[2] defined for normal blood pressure values without any antihypertensive drugs[a,b,c].
6.2	Decrease of body weight	RCT	Patients aged 13-17 years, with obesity +/- diabetes; studies, with duration≥ 52 weeks, performed on patients with BMI>30 Kg/m^2. BMI, percentage of weigh lost, and percentage of fat mass at endpoint[a,b,c].
6.3	Reduction of all-cause mortality	RCT	Patients aged 13-17 years, with obesity +/- diabetes; studies, with duration≥ 52 weeks, performed on patients with BMI >30 Kg/m^2. Mortality at endpoint[a,b,c].
6.4	Improvement of quality of life	RCT	Patients aged 13-17 years, with obesity +/- diabetes; studies, with duration≥ 52 weeks, performed on patients with BMI >30 Kg/m^2. Quality of life measured with any questionnaire at endpoint[a,c].
6.5	Perioperative mortality	RCT	Patients aged 13-17 years, with obesity +/- diabetes; studies, with duration≥ 52 weeks, performed on patients with BMI >30 Kg/m^2. Mortality during surgical procedures or hospital stay.
6.6	Perioperative surgical complications	RCT	Patients aged 13-17 years, with obesity +/- diabetes; studies, with duration≥ 52 weeks, performed on patients with BMI >30 Kg/m^2. Surgical complications during surgical procedures or hospital stay.
6.7	Serious adverse events (surgical and nonsurgical)	RCT	Patients aged 13-17 years, with obesity +/- diabetes; studies, with duration≥ 52 weeks, performed on patients with BMI>30 Kg/m^2. Any serious adverse events during surgical procedures or during follow-up at endpoint.
7.1	Obesity-related complications remission	RCT	Patients aged 60+ years, with obesity and at least one comorbid condition; studies, with duration≥ 52 weeks, performed on patients with BMI>35 or BMI>40 Kg/m^2. Remission for obstructive sleep apnea is defined as 1) C-PAP discontinuation[3], 2) symptoms

**N***	**Outcome**	**Type of study**	**Main inclusion criteria**

			remission (with specific questionnaires) or 3) Apnea-Hypopnea Index< 5 events/h at polysomnography[3]. Remission of dyslipidemia is defined as normal values of cholesterol without drugs[2]. Remission for hypertension[2] defined for normal blood pressure values without any antihypertensive drugs[a,b,c,d].
7.2	Decrease of body weight	RCT	Patients aged 60+ years, with obesity +/- diabetes; studies, with duration≥ 52 weeks, performed on patients with BMI>30 Kg/m². BMI, percentage of weigh lost, and percentage of fat mass at endpoint[a,b,c].
7.3	Reduction of all-cause mortality	RCT	Patients aged 60+ years, with obesity +/- diabetes; studies, with duration≥ 52 weeks, performed on patients with BMI >30 Kg/m². Mortality at endpoint[a,b,c].
7.4	Improvement of quality of life	RCT	Patients aged 60+ years, with obesity +/- diabetes; studies, with duration≥ 52 weeks, performed on patients with BMI >30 Kg/m². Quality of life measured with any questionnaire at endpoint.
7.5	Perioperative mortality	RCT	Patients aged 60+ years, with obesity +/- diabetes; studies, with duration≥ 52 weeks, performed on patients with BMI >30 Kg/m². Mortality during surgical procedures or hospital stay.
7.6	Perioperative surgical complications	RCT	Patients aged 60+ years, with obesity +/- diabetes; studies, with duration≥ 52 weeks, performed on patients with BMI >30 Kg/m². Surgical complications during surgical procedures or hospital stay.
7.7	Serious adverse events (surgical and nonsurgical)	RCT	Patients aged 60+ years, with obesity +/- diabetes; studies, with duration≥ 52 weeks, performed on patients with BMI>30 Kg/m2. Any serious adverse events during surgical procedures or during follow-up at endpoint.
8.1	Reduction of the incidence of Barrett disease	RCT	Patients aged 18+ years, with obesity; studies, with duration≥ 52 weeks, performed on patients with BMI>30 Kg/m² and diagnosis of gastroesophageal reflux disease. Barrett disease at endpoint.
8.2	Reduction of the incidence of gastroesophageal malignancies	RCT	Patients aged 18+ years with obesity; studies, with duration≥ 52 weeks, performed on patients with BMI>30 Kg/m² and diagnosis of gastroesophageal reflux disease. Gastro-esophageal cancer at endpoint.
8.3	Decrease of body weight	RCT	Patients aged 18+ years, with obesity; studies, with duration≥ 52 weeks, performed on patients with BMI>30 Kg/m² and diagnosis of gastroesophageal reflux disease. BMI, percentage of weigh lost, and percentage of fat mass at endpoint[a,b,c].

8.4	Improvement of quality of life	RCT	Patients aged 18+ years, with obesity; studies, with duration≥ 52 weeks, performed on patients with BMI>30 Kg/m^2 and diagnosis of gastroesophageal reflux disease. Quality of life measured with any questionnaire at endpoint.

N*	Outcome	Type of study	Main inclusion criteria
8.5	Perioperative mortality	RCT	Patients aged 18+ years, with obesity; studies, with duration≥ 52 weeks, performed on patients with BMI>30 Kg/m^2 and diagnosis of gastroesophageal reflux disease. Mortality during surgical procedures or hospital stay.
8.6	Perioperative surgical complications	RCT	Patients aged 18+ years, with obesity; studies, with duration≥ 52 weeks, performed on patients with BMI>30 Kg/m^2 and diagnosis of gastroesophageal reflux disease. Surgical complications during surgical procedures or hospital stay.
9.1	Reduction of hospital stay	RCT	Patients aged 18+ years, with obesity; studies, with duration≥ 52 weeks, performed on patients with BMI>30 Kg/m^2 and diagnosis of arthropathy. Hospital stay is defined as the number of days in hospital.
9.2	Reduction of all-cause mortality	RCT	Patients aged 18+ years, with obesity; studies, with duration≥ 52 weeks, performed on patients with BMI>30 Kg/m^2 and diagnosis of arthropathy. Mortality for any cause at endpoint.
9.3	Decrease of body weight (BMI; percentage of weigh lost; percentage of fat mass)	RCT	Patients aged 18+ years, with obesity; studies, with duration≥ 52 weeks, performed on patients with BMI>30 Kg/m^2 and diagnosis of arthropathy. BMI, percentage of weigh lost, and percentage of fat mass at endpoint
9.4	Reduction of rehospitalization	RCT	Patients aged 18+ years, with obesity; studies, with duration≥ 52 weeks, performed on patients with BMI>30 Kg/m^2 and diagnosis of arthropathy. Re-hospitalization within 30 days.
9.5	Reduction of orthopedic perioperative surgical complications	RCT	Patients aged 18+ years, with obesity; studies, with duration≥ 52 weeks, performed on patients with BMI>30 Kg/m^2 and diagnosis of arthropathy. Orthopedic surgical complications during surgical procedures or hospital stay.
9.6	Improvement of quality of life	RCT	Patients aged 18+ years, with obesity; studies, with duration≥ 52 weeks, performed on patients with BMI>30 Kg/m^2 and diagnosis of arthropathy. Quality of life measured with any questionnaire at endpoint.

N*	Outcome	Type of study	Main inclusion criteria
9.7	Perioperative surgical (bariatric) complications	RCT	Patients aged 18+ years, with obesity; studies, with duration≥ 52 weeks, performed on patients with BMI>30 Kg/m^2 and diagnosis of arthropathy. Surgical complications during surgical procedures or hospital stay.
9.8	Perioperative mortality	RCT	Patients aged 18+ years, with obesity; studies, with duration≥ 52 weeks, performed on patients with BMI>30 Kg/m^2 and diagnosis of arthropathy. Mortality for any cause during surgical procedures or hospital stay.
9.9	Serious adverse events (surgical and nonsurgical)	RCT	Patients aged 18+ years, with obesity; studies, with duration≥ 52 weeks, performed on patients with BMI>30 Kg/m^2 and diagnosis of arthropathy. Any serious adverse events during surgical procedures or during follow-up, at endpoint.
10.1	Increase of transplantation eligibility	RCT	Patients aged 18+ years, with obesity; studies, with duration≥ 52 weeks, performed on patients with BMI>30 Kg/m^2 and on a waiting list for renal/hepatic transplantation. Eligibility for transplantation after bariatric surgery.
10.2	Reduction of surgical (transplantation) complications	RCT	Patients aged 18+ years, with obesity; studies, with duration≥ 52 weeks, performed on patients with BMI>30 Kg/m^2 and on a waiting list for renal/hepatic transplantation. Surgical complications during transplantation.
10.3	Decrease of graft rejection	RCT	Patients aged 18+ years, with obesity; studies, with duration≥ 52 weeks, performed on patients with BMI>30 Kg/m^2 and on a waiting list for renal/hepatic transplantation. Graft rejection after transplantation.
10.4	Perioperative surgical (bariatric) complications	RCT	Patients aged 18+ years, with obesity; studies, with duration≥ 52 weeks, performed on patients with BMI>30 Kg/m^2 and on a waiting list for renal/hepatic transplantation. Surgical complications during surgical procedures or hospital stay.
10.5	Serious adverse events (surgical and nonsurgical)	RCT	Patients aged 18+ years, with obesity; studies, with duration≥ 52 weeks, performed on patients with BMI>30 Kg/m^2 and on a waiting list for renal/hepatic transplantation. Any serious adverse events during surgical procedures or during follow-up, at endpoint.
11.1	Reduction of incident malignancies	RCT	Patients aged 18+ years, with obesity; studies, with duration≥ 52 weeks, performed on patients with BMI>30 Kg/m^2. Any form of cancer at endpoint.

N*	Outcome	Type of study	Main inclusion criteria
11.2	Reduction of mortality for cancer	RCT	Patients aged 18+ years, with obesity; studies, with duration≥ 52 weeks, performed on patients with BMI>30 Kg/m^2. Cancer-related mortality at endpoint.
	**B. PERI-OPERATIVE WORK-UP/MANAGEMENT**		
12.1	Improvement of apneahypopnea index	RCT	Patients aged 18+ years, with obesity; studies, with duration≥ 52 weeks, performed on patients with BMI>30 Kg/m^2. Comparison between studies performing OSAS screening and studies not performing any screening. Apnea-hypopnea index at endpoint.
12.2	Reduction of perioperative mortality	RCT	Patients aged 18+ years, with obesity; studies, with duration≥ 52 weeks, performed on patients with BMI>30 Kg/m^2. Comparison between studies performing OSAS screening and studies not performing any screening. Mortality during surgical procedures or hospital stay.
12.5	Perioperative surgical complications	RCT	Patients aged 18+ years, with obesity; studies, with duration≥ 52 weeks, performed on patients with BMI>30 Kg/m^2. Comparison between studies performing OSAS screening and studies not performing any screening. Surgical complications during surgical procedures or hospital stay.
13.1	Improvement of apneahypopnea index	RCT	Patients aged 18+ years, with obesity and OSAS; studies, with duration≥ 52 weeks, performed on patients with BMI>30 Kg/m^2. Apnea-hypopnea index at endpoint[e].
13.2	Decrease of perioperative surgical complications	RCT	Patients aged 18+ years, with obesity and OSAS; studies, with duration≥ 52 weeks, performed on patients with BMI>30 Kg/m^2. Surgical complications during surgical procedures or hospital stay[e].
13.3	Reduction of perioperative mortality	RCT	Patients aged 18+ years, with obesity and OSAS; studies, with duration≥ 52 weeks, performed on patients with BMI>30 Kg/m^2. Mortality for any cause during surgical procedures or hospital stay[e].
14.1	Reduction of surgical dehiscence	RCT	Patients aged 18+ years, with obesity; studies, with duration≥ 52 weeks, performed on patients with BMI>30 Kg/m^2. Head-to-head comparisons and comparison between studies performing preoperative gastroscopy and studies not performing any screening[f]. Surgical dehiscence during hospital stay.

14.2	Reduction of reintervention	RCT	Patients aged 18+ years, with obesity; studies, with duration≥ 52 weeks, performed on patients with BMI>30 Kg/m^2. Head-to-head comparisons and comparison between studies performing preoperative gastroscopy and studies not performing any screening[f]. Re-intervention during follow-up.
14.3	Reduction of all-cause mortality	RCT	Patients aged 18+ years, with obesity; studies, with duration≥ 52 weeks, performed on patients with BMI>30 Kg/m^2. Head-to-head comparisons and comparison between studies performing preoperative gastroscopy and studies not performing any screening[f]. Mortality for any cause at endpoint.
14.4	Perioperative surgical complications	RCT	Patients aged 18+ years, with obesity; studies, with duration≥ 52 weeks, performed on patients with BMI>30 Kg/m^2. Head-to-head comparisons and comparison between studies performing preoperative gastroscopy and studies not performing any screening[f]. Surgical complications during surgical procedures or hospital stay.
15.1	Reduction of perioperative surgical complications	RCT	Patients aged 18+ years, with obesity; studies, with duration≥ 52 weeks, performed on patients with BMI>30 Kg/m^2 and reporting information on pre-surgical weight-loss programs[g]. Surgical complications during surgical procedures or hospital stay.
15.2	Reduction of length of surgical procedures	RCT	Patients aged 18+ years, with obesity; studies, with duration≥ 52 weeks, performed on patients with BMI>30 Kg/m^2 and reporting information on pre-surgical weight-loss programs[g]. Duration of surgical procedure.

N*	Outcome	Type of study	Main inclusion criteria
15.3	Decrease of body weight (BMI; percentage of weigh lost; percentage of fat mass)	RCT	Patients aged 18+ years, with obesity; studies, with duration≥ 52 weeks, performed on patients with BMI>30 Kg/m^2 and reporting information on pre-surgical weight-loss programs[g]. BMI, percentage of weigh lost, and percentage of fat mass at endpoint (after surgery).
15.4	Improvement of quality of life	RCT	Patients aged 18+ years, with obesity; studies, with duration≥ 52 weeks, performed on patients with BMI>30 Kg/m^2 and reporting information on pre-surgical weight-loss programs[g]. Quality of life measured with any questionnaire at endpoint.

16.1	Reduction of perioperative mortality	RCT	Patients aged 18+ years, with obesity; studies, with duration≥ 52 weeks, performed on patients with BMI>30 Kg/m^2. Head-to-head comparisons and comparisons between studies using pre-operative anticoagulants and studies not using anticoagulants[h]. Mortality for any cause at endpoint.
16.2	Reduction of surgical complications	RCT	Patients aged 18+ years, with obesity; studies, with duration≥ 52 weeks, performed on patients with BMI>30 Kg/m^2. Head-to-head comparisons and comparisons between studies using pre-operative anticoagulants and studies not using anticoagulants[h]. Surgical complications during surgical procedures or hospital stay.
16.3	Reduction of thromboembolic complications	RCT	Patients aged 18+ years, with obesity; studies, with duration≥ 52 weeks, performed on patients with BMI>30 Kg/m^2. Head-to-head comparisons and comparisons between studies using pre-operative anticoagulants and studies not using anticoagulants[h]. Thromboembolic complications during surgical procedures or hospital stay.
16.5	Increase of bleeding	RCT	Patients aged 18+ years, with obesity; studies, with duration≥ 52 weeks, performed on patients with BMI>30 Kg/m^2. Head-to-head comparisons and comparisons between studies using pre-operative anticoagulants and studies not using anticoagulants[h]. Bleeding during surgical procedures or hospital stay.
17.1	Reduction of perioperative infective complications	RCT	Patients aged 18+ years, with obesity; studies, with duration≥ 52 weeks, performed on patients with BMI>30 Kg/m^2. Head-to-head comparisons and comparisons between studies using pre-operative antibiotics and studies not using antibiotics[i]. Infective complications during surgical procedures or hospital stay.
17.2	Reduction of perioperative mortality	RCT	Patients aged 18+ years, with obesity; studies, with duration≥ 52 weeks, performed on patients with BMI>30 Kg/m^2. Head-to-head comparisons and comparisons between studies using pre-operative antibioitcs and studies not using antibioitcs [i]. Mortality for any cause at endpoint.
**N***	**Outcome**	**Type of study**	**Main inclusion criteria**
17.3	Reduction of perioperative surgical complications	RCT	Patients aged 18+ years, with obesity; studies, with duration≥ 52 weeks, performed on patients with BMI>30 Kg/m^2. Head-to-head comparisons and comparisons between studies using pre-operative antibioitcs and studies not using antibioitcs [i]. Surgical complications during surgical procedures or hospital stay.

17.4	Reduction of hospital stay	RCT	Patients aged 18+ years, with obesity; studies, with duration≥ 52 weeks, performed on patients with BMI>30 Kg/m^2. Head-to-head comparisons and comparisons between studies using pre-operative antibioitcs and studies not using antibioitcs [i]. Duration of hospital stay.
18.1	Reduction of perioperative surgical complications	RCT	Patients aged 18+ years, with obesity; studies, with duration≥ 52 weeks, performed on patients with BMI>30 Kg/m^2. Head-to-head comparisons and comparisons between studies using and not using ERABS. Surgical complications during surgical procedures or hospital stay.
18.2	Reduction of time to patient mobilization	RCT	Patients aged 18+ years, with obesity; studies, with duration≥ 52 weeks, performed on patients with BMI>30 Kg/m^2. Head-to-head comparisons and comparisons between studies using and not using ERABS. Time needed for patients mobilization (days).
18.3	Reduction of postsurgical pain	RCT	Patients aged 18+ years, with obesity; studies, with duration≥ 52 weeks, performed on patients with BMI>30 Kg/m^2. Head-to-head comparisons and comparisons between studies using and not using ERABS. Pain assessed with any questionnaire, after surgical procedure and/or during hospital stay.
18.4	Reduction of hospital stay	RCT	Patients aged 18+ years, with obesity; studies, with duration≥ 52 weeks, performed on patients with BMI>30 Kg/m^2. Head-to-head comparisons and comparisons between studies using and not using ERABS. Duration of hospital stay.
18.5	Reduction of time for enteral feeding/hydration	RCT	Patients aged 18+ years, with obesity; studies, with duration≥ 52 weeks, performed on patients with BMI>30 Kg/m^2. Head-to-head comparisons and comparisons between studies using and not using ERABS. Time needed for enteral feeding/hydration (hours).
18.6	Reduction of all-cause mortality	RCT	Patients aged 18+ years, with obesity; studies, with duration≥ 52 weeks, performed on patients with BMI>30 Kg/m^2. Head-to-head comparisons and comparisons between studies using and not using ERABS. Mortality for any cause at endpoint.
18.7	Increase of quality of life	RCT	Patients aged 18+ years, with obesity; studies, with duration≥ 52 weeks, performed on patients with BMI>30 Kg/m^2. Head-to-head comparisons and comparisons between studies using and not using ERABS. Quality of life measured with any questionnaire at endpoint.

N*	Outcome	Type of study	Main inclusion criteria

19.1	Increase of 25-OH vitamin D	RCT	Patients aged 18+ years, with obesity; studies, with duration≥ 24 weeks, performed on patients with BMI>30 Kg/m[2] and reporting information on vitamin/protein supplementation prior surgical procedures. 25-OH vitamin D levels at endpoint.
20.1	Reduction of incident gallbladder stones	RCT	Patients aged 18+ years, with obesity; studies, with duration≥ 24 weeks, performed on patients with BMI>30 Kg/m[2] and reporting information on ursodeoxycholic acid supplementation prior surgical procedures. Incident gallbladder stones at endpoint.
20.2	Reduction of cholecystectomy	RCT	Patients aged 18+ years, with obesity; studies, with duration≥ 24 weeks, performed on patients with BMI>30 Kg/m[2] and reporting information on ursodeoxycholic acid supplementation prior surgical procedures. Incident cholecystectomy at endpoint.
21.1,22.1, 23.1,24.1,25.1	Diabetes remission	RCT	Patients aged 18+ years, with obesity and diabetes (or subgroups with diabetes); studies performed on patients with BMI 30-34.9, >35, and >40 Kg/m[2] and duration≥ 52 weeks[a,b,c]. A network metanalysis will be performed to compare the efficacy and safety of individual surgical procedures. Diabetes remission[1] will be evaluated yearly, whenever possible, and at endpoint.
21.2,22.2, 23.2,24.2, 25.2	Improvement of glycometabolic control	RCT	Patients aged 18+ years, with obesity +/- diabetes; studies performed on patients with BMI 30-34.9, >35, and >40 Kg/m[2] and duration≥ 52 weeks. A network metanalysis will be performed to compare the efficacy and safety of individual surgical procedures. HbA1c; FPG; lipid profile; blood pressure at endpoint[a,b,c].
21.3,22.3,23.3,24.3,25.3	Decrease of body weight	RCT	Patients aged 18+ years, with obesity +/- diabetes; studies performed on patients with BMI 30-34.9, >35, and >40 Kg/m[2] and duration≥ 52 weeks. A network metanalysis will be performed to compare the efficacy and safety of individual surgical procedures. BMI, percentage of weigh lost, and percentage of fat mass at endpoint[a,b,c].
21.4,22.4,23.4,24.4,25.4	Reduction of macrovascular complications	RCT	Patients aged 18+ years, with obesity +/- diabetes; studies performed on patients with BMI 30-34.9, >35, and >40 Kg/m[2] and duration≥ 52 weeks. A network metanalysis will be performed to compare the efficacy and safety of individual surgical procedures. Nonfatal myocardial infarction, nonfatal stroke e cardiovascular mortality (composite endpoint) at endpoint[a,b,c].
21.5,22.5,23.5,24.5,25.5	Reduction of all-cause mortality	RCT	Patients aged 18+ years, with obesity +/- diabetes; studies performed on patients with BMI 30-34.9, >35, and >40 Kg/m2 and duration≥ 52 weeks[a,b,c]. A network metanalysis will be performed to compare the efficacy and safety of individual surgical procedures. Mortality at endpoint.

N*	Outcome	Type of study	Main inclusion criteria
21.6,22.6,23.6,24.6,25.6	Improvement of quality of life	RCT	Patients aged 18+ years, with obesity +/- diabetes; studies performed on patients with BMI 30-34.9, >35, and >40 Kg/m^2 and duration≥ 52 weeks. A network metanalysis will be performed to compare the efficacy and safety of individual surgical procedures. Quality of life measured with any questionnaire at endpoint[a,c].
24.7, 25.7	Hypertension remission	RCT	Patients aged 18+ years, with obesity and at least one comorbid condition; studies performed on patients with BMI>35 or BMI>40 Kg/m^2, and duration≥ 52 weeks. A network metanalysis will be performed to compare the efficacy and safety of individual surgical procedures. Remission is defined as normal blood pressure values without any antihypertensive drugs[2].
24.8, 25.8	Obesity-related complications remission	RCT	Patients aged 18+ years, with obesity and at least one comorbid condition; studies, with duration≥ 52 weeks, performed on patients with BMI>35 or BMI>40 Kg/m^2. A network metanalysis will be performed to compare the efficacy and safety of individual surgical procedures. Remission for obstructive sleep apnea is defined as 1) C-PAP discontinuation[3], 2) symptoms remission (with specific questionnaires) or 3) Apnea-Hypopnea Index< 5 events/h at polysomnography[3]. Remission of dyslipidemia is defined as normal values of cholesterol without drugs[2]. Remission for hypertension[2] defined for normal blood pressure values without any antihypertensive drugs[a,b,c].
21.7,22.7,23.7,24.9,25.9	Perioperative mortality	RCT	Patients aged 18+ years, with obesity +/- diabetes; studies, with duration≥ 52 weeks, performed on patients with BMI 30-34.9, >35, and >40 Kg/m^2. A network metanalysis will be performed to compare the efficacy and safety of individual surgical procedures. Mortality during surgical procedures or hospital stay.
21.8,22.8,23.8,24.10,25.10	Perioperative surgical complications	RCT	Patients aged 18+ years, with obesity +/- diabetes; studies, with duration≥ 52 weeks, performed on patients with BMI 30-34.9, >35, and >40 Kg/m^2. A network metanalysis will be performed to compare the efficacy and safety of individual surgical procedures. Surgical complications during surgical procedures or hospital stay.
21.9,22.9,23.9,24.121,25.11	Serious adverse events (surgical and non-surgical)	RCT	Patients aged 18+ years, with obesity +/- diabetes; studies, with duration≥ 52 weeks, performed on patients with BMI 30-34.9, >35, and >40 Kg/m^2. A network metanalysis will be performed to compare the efficacy and safety of individual surgical procedures. Any serious adverse events during surgical procedures or during follow-up at endpoint.

26.1	Diabetes remission	RCT	Patients aged 18+ years, with obesity and diabetes (or subgroups with diabetes); studies, with duration≥ 52 weeks, performed on patients with BMI>30 Kg/m^2 undergoing endoscopic surgical

N*	Outcome	Type of study	Main inclusion criteria
			procedures[b,c]; diabetes remission[1] will be evaluated yearly whenever possible, and at endpoint.
26.2	Improvement of glycometabolic control	RCT	Patients aged 18+ years, with obesity +/- diabetes (or subgroups with diabetes); studies, with duration≥ 52 weeks, performed on patients with BMI>30 Kg/m^2 undergoing endoscopic surgical procedures[b,c]. HbA1c; FPG; lipid profile; blood pressure at endpoint.
26.3	Decrease of body weight	RCT	P Patients aged 18+ years, with obesity; studies, with duration≥ 52 weeks, performed on patients with BMI>30 Kg/m^2 undergoing endoscopic surgical procedures[b,c]. BMI, percentage of weigh lost, and percentage of fat mass at endpoint.
26.4	Reduction of macrovascular complications	RCT	Patients aged 18+ years, with obesity; studies, with duration≥ 52 weeks, performed on patients with BMI>30 Kg/m^2 undergoing endoscopic surgical procedures[b,c]. Nonfatal myocardial infarction, nonfatal stroke e cardiovascular mortality (composite endpoint) at endpoint.
26.5	Reduction of all-cause mortality	RCT	Patients aged 18+ years, with obesity; studies, with duration≥ 52 weeks, performed on patients with BMI>30 Kg/m^2 undergoing endoscopic surgical procedures[b,c]. Mortality at endpoint.
26.6	Improvement of quality of life	RCT	Patients aged 18+ years, with obesity; studies, with duration≥ 52 weeks, performed on patients with BMI>30 Kg/m^2 undergoing endoscopic surgical procedures[b,c]. Quality of life measured with any questionnaire at endpoint.
26.7	Perioperative mortality	RCT	Patients aged 18+ years, with obesity; studies, with duration≥ 52 weeks, performed on patients with BMI >30 Kg/m^2. Mortality during surgical procedures or hospital stay.
26.8	Perioperative surgical complications	RCT	Patients aged 13-17 years, with obesity; studies, with duration≥ 52 weeks, performed on patients with BMI >30 Kg/m^2. Surgical complications during surgical procedures or hospital stay.
26.9	Serious adverse events (surgical and nonsurgical)	RCT	Patients aged 18+ years, with obesity; studies, with duration≥ 52 weeks, performed on patients with BMI>30 Kg/m^2. Any serious adverse events during surgical procedures or during follow-up at endpoint.

27.1, 28.1	Prevention of incidence/recurrence of diabetes	RCT	Patients aged 18+ years, with obesity, undergoing bariatric surgery with postsurgical weight regain; studies, with duration≥ 52 weeks. Incident diabetes or recurrence[1].
27.2, 28.2	Improvement of glycometabolic control (HbA1c; FPG; lipid profile; blood pressure)	RCT	Patients aged 18+ years, with obesity, undergoing bariatric surgery with postsurgical weight regain; studies, with duration≥ 52 weeks. HbA1c; FPG; lipid profile; blood pressure at endpoint.

N*	Outcome	Type of study	Main inclusion criteria
27.3, 28.3	Decrease of body weight (BMI; percentage of weigh lost; percentage of fat mass)	RCT	Patients aged 18+ years, with obesity, undergoing bariatric surgery with postsurgical weight regain; studies, with duration≥ 52 weeks. BMI, percentage of weigh lost, and percentage of fat mass at endpoint.
27.4, 28.4	Reduction of macrovascular complications	RCT	Patients aged 18+ years, with obesity, undergoing bariatric surgery with postsurgical weight regain; studies, with duration≥ 52 weeks. Nonfatal myocardial infarction, nonfatal stroke e cardiovascular mortality (composite endpoint) at endpoint.
27.5, 28.5	Reduction of all-cause mortality	RCT	Patients aged 18+ years, with obesity, undergoing bariatric surgery with postsurgical weight regain; studies, with duration≥ 52 weeks. Mortality at endpoint.
27.6, 28.6	Improvement of quality of life	RCT	Patients aged 18+ years, with obesity, undergoing bariatric surgery with postsurgical weight regain; studies, with duration≥ 52 weeks. Quality of life measured with any questionnaire at endpoint.
27.7, 28.7	Perioperative mortality	RCT	Patients aged 18+ years, with obesity, undergoing bariatric surgery with postsurgical weight regain; studies, with duration≥ 52 weeks. Mortality during surgical procedures or hospital stay.
27.8, 28.8	Perioperative surgical complications	RCT	Patients aged 18+ years, with obesity, undergoing bariatric surgery with postsurgical weight regain; studies, with duration≥ 52 weeks. Surgical complications during surgical procedures or hospital stay.
27.9, 28.9	Serious adverse events (surgical and nonsurgical)	RCT	Patients aged 18+ years, with obesity, undergoing bariatric surgery with postsurgical weight regain; studies, with duration≥ 52 weeks. Any serious adverse events during surgical procedures or during follow-up at endpoint
29.1	Prevention of incidence/recurrence of diabetes	RCT	Patients aged 18+ years, with obesity, undergoing bariatric surgery with postsurgical treatment with drugs approved for obesity; studies, with duration≥ 52 weeks. Incident diabetes or recurrence[1].
29.2	Improvement of glycometabolic control (HbA1c; FPG; lipid profile; blood pressure)	RCT	Patients aged 18+ years, with obesity, undergoing bariatric surgery with postsurgical treatment with drugs approved for obesity; studies, with duration≥ 52 weeks. HbA1c; FPG; lipid profile; blood pressure at endpoint.

29.3	Decrease of body weight (BMI; percentage of weigh lost; percentage of fat mass)	RCT	Patients aged 18+ years, with obesity, undergoing bariatric surgery with postsurgical treatment with drugs approved for obesity; studies, with duration≥ 52 weeks. BMI, percentage of weigh lost, and percentage of fat mass at endpoint.
29.4	Reduction of macrovascular complications	RCT	Patients aged 18+ years, with obesity, undergoing bariatric surgery with postsurgical treatment with drugs approved for obesity; studies, with duration≥ 52 weeks. Nonfatal myocardial infarction, nonfatal stroke e cardiovascular mortality (composite endpoint) at endpoint.

**N***	**Outcome**	**Type of study**	**Main inclusion criteria**
29.5	Reduction of all-cause mortality	RCT	Patients aged 18+ years, with obesity, undergoing bariatric surgery with postsurgical treatment with drugs approved for obesity; studies, with duration≥ 52 weeks. Mortality at endpoint.
29.6	Improvement of quality of life	RCT	Patients aged 18+ years, with obesity, undergoing bariatric surgery with postsurgical treatment with drugs approved for obesity; studies, with duration≥ 52 weeks. Quality of life measured with any questionnaire at endpoint.
29.7	Perioperative mortality	RCT	Patients aged 18+ years, with obesity, undergoing bariatric surgery with postsurgical treatment with drugs approved for obesity; studies, with duration≥ 52 weeks. Mortality during surgical procedures or hospital stay.
29.8	Perioperative surgical complications	RCT	Patients aged 18+ years, with obesity, undergoing bariatric surgery with postsurgical treatment with drugs approved for obesity; studies, with duration≥ 52 weeks. Surgical complications during surgical procedures or hospital stay.
29.9	Serious adverse events (surgical and nonsurgical)	RCT	Patients aged 18+ years, with obesity, undergoing bariatric surgery with postsurgical treatment with drugs approved for obesity; studies, with duration≥ 52 weeks. Any serious adverse events during surgical procedures or during follow-up at endpoint
30.1, 31.1	Prevention of incidence/recurrence of diabetes	RCT	Patients aged 18+ years, with obesity, undergoing bariatric surgery and postsurgical multidisciplinary follow-up/life style modification; studies, with duration≥ 52 weeks. Incident diabetes or recurrence[1].
30.2, 31.2	Improvement of glycometabolic control (HbA1c; FPG; lipid profile; blood pressure)	RCT	Patients aged 18+ years, with obesity, undergoing bariatric surgery with postsurgical treatment with drugs approved for obesity; studies, with duration≥ 52 weeks. HbA1c; FPG; lipid profile; blood pressure at endpoint.

30.3, 31.3	Decrease of body weight (BMI; percentage of weigh lost; percentage of fat mass)	RCT	Patients aged 18+ years, with obesity, undergoing bariatric surgery with postsurgical treatment with drugs approved for obesity; studies, with duration≥ 52 weeks. Nonfatal myocardial infarction, nonfatal stroke e cardiovascular mortality (composite endpoint) at endpoint.
30.4, 31.4	Reduction of weight regain	RCT	Patients aged 18+ years, with obesity, undergoing bariatric surgery with postsurgical treatment with drugs approved for obesity; studies, with duration≥ 52 weeks. Percentage of weight regain.
30.5, 31.5	Improvement of quality of life	RCT	Patients aged 18+ years, with obesity, undergoing bariatric surgery with postsurgical treatment with drugs approved for obesity; studies, with duration≥ 52 weeks. Quality of life measured with any questionnaire at endpoint

N*	Outcome	Type of study	Main inclusion criteria
32.1	Reduction of cesarean delivery	RCT	Women aged 18+ years, undergoing bariatric surgery and planning pregnancy; studies, with duration≥ 24 weeks. Incidence of cesarean delivery.
32.2	Reduction of pre-term delivery	RCT	Women aged 18+ years, undergoing bariatric surgery and planning pregnancy; studies, with duration≥ 24 weeks. Incidence of preterm delivery (before 37 weeks)[4].

*See outcomes of Table 3S. OSAS: Obstructive Sleep Apnea Syndrome; C-PAP: Continuous-Positive Airway Pressure; ERABS: Enhanced Recovery After Bariatric Surgery.

a. Sensitivity analyses will be performed including trials with mean BMI at enrolment between 30 and 35, >35, and >40, whenever possible.

b. Metaregression analyses will be performed on all trials, irrespective of BMI at entry, plotting the effect on outcome versus mean BMI at enrolment, in order to obtain a further estimate of the range of effect on outcome in patients with BMI within the defined range.

c. Subgroup analyses for studies with duration> 156 weeks and >260 weeks, and different comparators (active or placebo/none) will be performed for all clinical questions, whenever possible.

d. Sensitivity analyses will be performed including trials with a mean age at enrolment≥ 60 years, whenever possible.

e. Metaregression analyses will be performed on all trials, irrespective of a diagnosis of OSAS as inclusion criteria (i.e. all RCTs reporting the proportion of patients with OSAS at entry), plotting the effect on outcome versus proportion of patients with OSAS at enrolment, in order to obtain a further estimate of the range of effect on outcome in patients with OSAS.

f. Sensitivity analyses will be performed by including trials not reporting information on pre-operative gastroscopy within the group of trials not performing gastroscopy.

g. Sensitivity analyses will be performed by including trials not reporting information on pre-operative weightloss programs within the group of trials not adopting pre-surgical weight-loss programs.

h. Sensitivity analyses will be performed by including trials not reporting information on pre-operative anticoagulant use within the group of trials not adopting pre-surgical anticoagulant use.

i. Sensitivity analyses will be performed by including trials not reporting information on pre-operative antibiotic use within the group of trials not adopting pre-surgical antibiotic use.
